# OD-ViTFScL: Asynchronous Few-Shot Continual Learning for Intelligent Process Monitoring and Fault Diagnosis in Petrochemical Plants

**DOI:** 10.3390/s26165283

**Published:** 2026-08-20

**Authors:** Oyekunle Oshidele, Sen Lin

**Affiliations:** Department of Computer Science, University of Houston, Houston, TX 77004, USA; slin50@central.uh.edu

**Keywords:** continual learning, machine learning, process monitoring, catastrophic forgetting, chemical plant

## Abstract

Petrochemical plants are complex facilities composed of interconnected equipment to produce essential products for daily human activities. In view of the adoption of the Industrial Internet of Things (IIoT), these facilities use various sensors, including those for level, flow, temperature, and pressure, to monitor and control operations. Several process faults can be detected and classified using data from these sensors. Detecting and classifying these faults helps mitigate production losses, improve product quality, decrease environmental concerns, and improve human safety. Conventional static learning, which learns from historical data, and continual learning, which learns incremental tasks with known preset boundaries, fall short on asynchronous online unknown streaming boundary tasks such as the OpenWorld Problem, which is the true representation of real-world dynamic plant scenarios. To tackle these challenges, we propose a novel online asynchronous framework termed OD-ViTFScL. Our framework utilizes a dual-attention backbone, which concatenates orthogonal information from attention on the vertical time-series data and horizontal inter-sensor relationships. The fused MLP from the temporal and sensor streams learns independent weights for each orthogonal axis. Our framework also uses ODIN, an out-of-distribution technique, to trigger the arrival of a new fault class in the streaming data. Extensive experiments on the Tennessee Eastman Process (TEP) and Case Western Reserve University (CWRU) bearing datasets validate that our proposed OD-ViTFScL outperforms other state-of-the-art fault detection and diagnosis (FDD) approaches.

## 1. Introduction

Modern petrochemical plants are complex facilities that are distributed across large geographical regions with several processing units. Each unit itself is a complex system consisting of many interacting physical and chemical components. To monitor these large operations, the IIoT is adopted, which requires sensors to be installed at several points of interest to observe and control process variables in the Distributed Control System (DCS) and other controllers such as the Programmable Logic Controller (PLC). Without proper detection of abnormalities in these processes and quick intervention by maintenance and operation technicians, an undesirable event or failure can occur. This could spread to other facilities and cause a significant unplanned plant shutdown, leading to a substantial financial loss or a safety event (fatality). In the context of Industry 4.0 [1,2], where petrochemical facilities are dynamic and complex [3,4,5,6], modeling the plant from first principles has become more challenging, and there is a need to design monitoring systems that can scale to thousands or even millions of sensors. Data-driven (machine learning) approaches have emerged as an efficient and scalable solution to the aforementioned complex environment. The main idea behind the data-driven approach is feature extraction, where the process data are transformed into lower-dimensional and more informative data. Several statistical data approaches such as dynamic principal component analysis (PCA) [7] and modified partial least squares (PLS) [8] have been utilized in the past. Neural network models such as DNN [9], CNN [10], and LSTM [11] have also been adopted for process fault detection and diagnosis (FDD) in batch chemical plant problem settings using historical datasets. However, these static approaches will largely fail in practical scenarios, where data streams sequentially—not only normal process data but also fault occurrences—and the data distributions can continuously shift. The distribution shifts can take various forms, such as dramatic changes when the types of faults change, or continuous shifts over time due to factors like operating condition changes or equipment aging, which is more challenging.  Naively adapting the static models with new data will lead to a notorious problem, i.e., catastrophic forgetting, where the previously learned knowledge will be forgotten during new knowledge learning [12]. To address this, continual learning (CL) has recently emerged in manufacturing industries [13,14,15], which aims to continuously adapt the model with new data without catastrophic forgetting. Despite these advances, existing studies that leverage continual learning techniques still suffer from key limitations such as class imbalance from few-shot faulty data, unknown task boundaries (the OpenWorld scenario), limited performance, etc. This calls for a new design of continual learning framework to address these limitations toward practical process monitoring and fault diagnosis in petrochemical plants. In this paper, our contributions are as follows:

1. Formulate petrochemical fault detection and diagnosis as an unknown boundary continual learning problem—the OpenWorld Problem.
2. Develop a novel framework, OD-ViTFScL, for fault detection and diagnosis in this setting.
3. Run experiments on two domain datasets with class imbalance and showcase the superior performance of our design compared to related baselines.

The remainder of this paper is organized as follows: Section 2 highlights other related fault detection and diagnosis (FDD) work. Section 3 formulates the problem. Section 4 describes the methods utilized. Section 5 showcases the experiments. Section 6 highlights the results, and Section 7 highlights the conclusions and future directions of our work.

## 2. Related Work

### 2.1. Static Learning

Many studies in the field of fault detection and diagnosis have explored static machine learning frameworks. In Ref. [9], fault detection and classification problems are tackled using a Multi-Layer Perceptron. The authors tested the effects of two hyperparameters: the number of hidden layers, and the number of neurons in the final hidden layer. They also examined the effect of input data augmentation on the accuracy of their models. Process monitoring and fault detection techniques have been developed for binary and continuous process variables [16]. Convolutional Neural Network (CNN): According to Ref. [10,14,17,18], the feature extraction capability of CNNs enables them to outperform regular deep neural network architectures. The paper showcases how a model with 11 convolutional layers for feature extraction followed by one fully connected and one softmax layer for classification can isolate 29 classes of TE process data (28 fault classes and one normal). CNNs have limitations in handling tabular data and providing interpretable results. To address these issues, Ref. [13] proposes Order-Invariant and Interpretable Dilated Convolution Neural Network (OIDLCNN-SHAP), which combines feature clustering, dilated convolutions, and SHAP explanations.

An autoencoder combined with LSTM is proposed in [11] for anomaly detection to identify rare fault events in chemical plants, where the autoencoder is trained on normal data to learn low-dimensional representations, enabling it to identify anomalies effectively. Hybrid frameworks combining signal processing with deep learning have demonstrated strong performance across diverse structural and industrial health monitoring applications, including GPR-based bridge deck delamination detection [19]. Transformer-based techniques have been deployed in aerospace to detect anomalies in aircraft fuel systems and mechanical bearing fault diagnosis [20,21]. Model ensemble methods with few-shot learning are adopted in the microservices domain in cloud computing [22]. A physics-constrained adaptive style transfer network built on multiscale wavelet transform is utilized in fault diagnosis [23].

### 2.2. Continual Learning

The aforementioned related works deal mostly with stationary datasets in chemical plants, which in the context of continual learning is referred to as a single task. Continual learning must deal with non-stationary dynamic datasets [24,25,26,27,28,29], where intelligent agents learn incremental tasks. According to Ref. [14], new fault classes are learned by prompting the base learning framework in an incremental hierarchical manner. Ref. [15] tackled online continual learning by proposing three critical methods: Retrospect Coreset Selection (RCS), which reduces redundant training and improves efficiency by selecting the most relevant data; Global Balance Technique (GBT), which ensures balanced coreset selection and robust model performance; and Confidence and Uncertainty-driven Pseudo-label Learning (CUPL), which updates the model using unlabeled data for continuous learning. Continual learning models experience significant fluctuations in performance when sequentially introduced classes exhibit inter-class similarity. In order to address this challenge, Ref. [30] dynamically groups classes into the minimum number of “Pseudo-tasks” based on prototype similarity, with independent classifiers trained for each pseudo-task. Despite these advances mentioned above, existing continual learning methods [31,32,33,34,35] applied to industrial fault diagnosis still exhibit critical limitations such as performance degradation due to insufficient feature extraction, imbalanced class distributions, and unknown task boundaries, which our approach—OD-ViTFScL, a novel online asynchronous continual learning framework—tackles. OD-ViTFScL handles these limitations by developing dual orthogonal (dual-attention) feature extraction and leveraging out-of-distribution techniques to trigger the arrival of a new fault in the unknown boundary task scenario, while registering few shots in class imbalance.

## 3. Problem Formulation

### 3.1. Fault Diagnosis Problem

Fault diagnosis in petrochemical plants refers to the detection and identification of abnormal operating conditions in which one or more process variables—including pressure, temperature, liquid level, flow rate, and valve position—deviate beyond their acceptable operating limits. Such deviations manifest as distinct fault modes, ranging from over-temperature events and high liquid levels to valve sticking, feed composition changes, and reactor cooling failures. Left undetected, these faults propagate across interconnected process units, leading to unplanned plant shutdowns, production losses, environmental hazards, and safety incidents.

### 3.2. Formulation as a Machine Learning Problem

The fault diagnosis problem is formulated as a multi-class classification problem. Given a window of multivariate sensor observations $X_{t}\in R^{d\times L}$ collected at time *t* across *d* sensor channels over a sliding window of length *L*, the objective is to learn a mapping $f_{\theta }:R^{d\times L}\to Y$ from the sensor space to a label space $Y=\{y_{0},y_{1},\ldots ,y_{C}\}$, where $y_{0}$ denotes the normal operating state and $y_{1},\ldots ,y_{C}$ denote *C* distinct fault classes. A labeled training dataset $D={\left\{\left(X_{i},y_{i}\right)\right\}}_{i=1}^{N}$ is constructed from historical plant records containing both normal operation and documented fault occurrences across all known fault types. The model $f_{\theta }$ is trained to minimize the classification loss over D.

### 3.3. Formulation as a Continual Learning Problem

Static machine learning formulations assume that the complete set of fault classes is known and available during training, which does not reflect real-world petrochemical plant operations, where new fault modes emerge over time as operating conditions evolve, equipment ages, or process configurations change. To address this limitation, we formulate the fault diagnosis problem as an asynchronous few-shot class-incremental continual learning problem, defined as follows:

The system operates over an ordered sequence of tasks $T=\{T_{0},T_{1},T_{2},\ldots \}$. Task $T_{0}$ is the base learning phase, during which the model is trained offline on a fully labeled historical dataset $D_{0}={\left\{\left(X_{i},y_{i}\right)\right\}}_{i=1}^{N}$ covering a set of known fault classes $C_{0}$, using standard supervised learning. All subsequent tasks $T_{k}$, k≥1, constitute the streaming phase, which differs from conventional continual learning in three critical respects that reflect real industrial conditions:

First, new fault classes $C_{k}\notin C_{k-1}$ may emerge at any point during plant operation, with no prior notification and no preset inter-task boundary—a setting that we term the OpenWorld Problem, distinguishing it from prior work that assumes known task boundaries. Second, when a novel fault is flagged, only *K* labeled support samples are available (K∈{1,5,10}), reflecting the realistic industrial constraint that fault occurrences are rare and expert annotation is costly and time-consuming. Third, the model must simultaneously perform in-distribution classification of all accumulated known fault classes and out-of-distribution detection of genuinely novel, as-yet-unseen fault modes, without access to any samples from previously learned tasks $T_{0},\ldots ,T_{k-1}$.

Formally, the accumulated class set at task $T_{k}$ is $C_{k}=C_{k-1}\cup \left\{C_{k}\right\}$. The classifier $f_{\theta }$ must correctly identify all classes in $C_{k}$ on future inputs while simultaneously recognizing inputs from genuinely novel fault modes as out-of-distribution.

## 4. Methods

**Figure 1:**
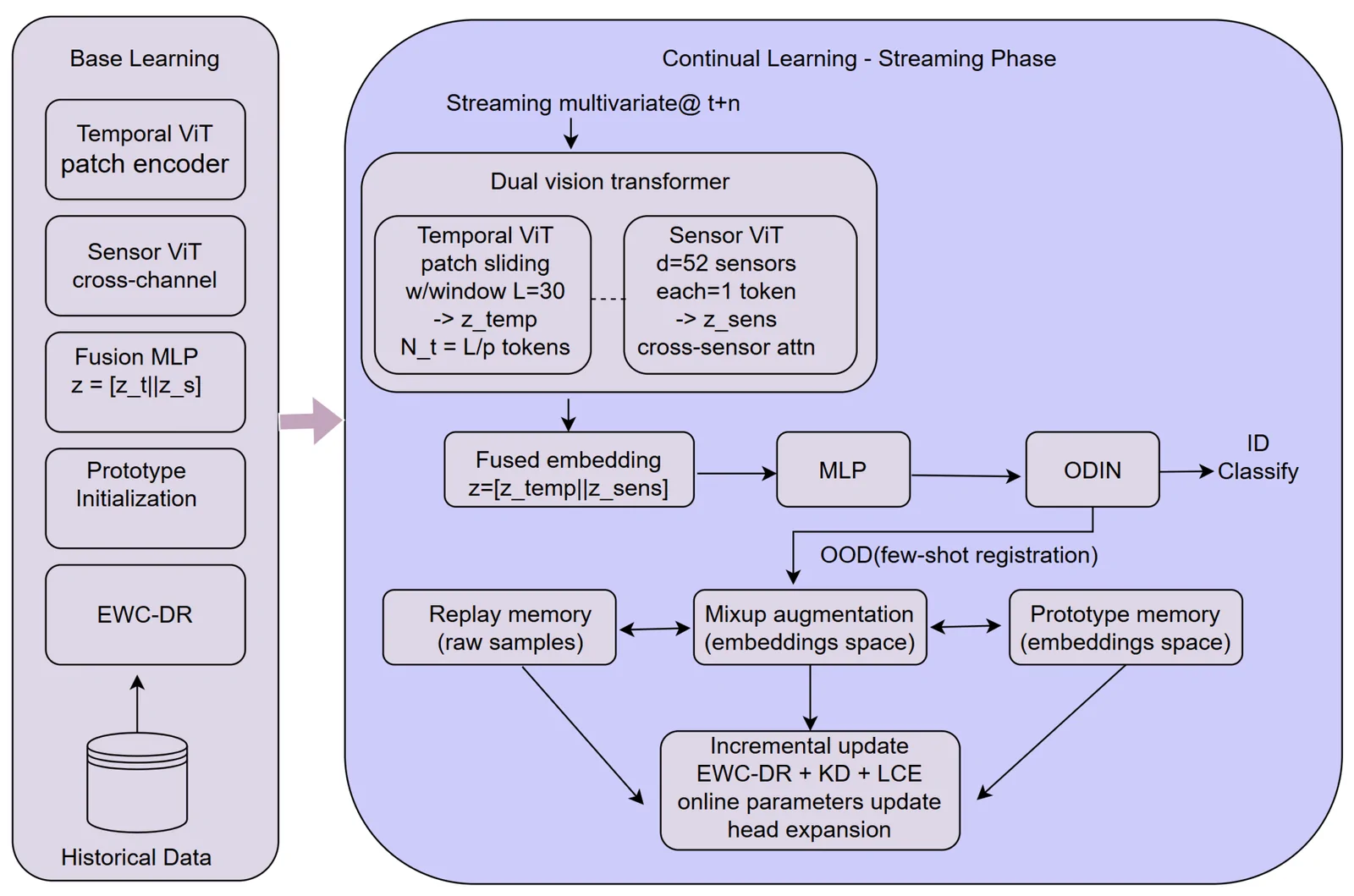
Our OD-ViTFScL architecture.

To address the challenges above, in this work we develop a novel framework OD-ViTFScL, as shown in Figure 1, which introduces three tightly integrated methods: First, a Dual Vision Transformer (ViT) [36] backbone—a Transformer-based image classification technique modified to process the multivariate sensor stream from two orthogonal axes simultaneously: a Temporal ViT that partitions the time axis into non-overlapping patches to capture sequential fault onset dynamics via patch-based self-attention [37,38], and a Sensor ViT that treats each of the *d* sensor channels as an independent token to capture cross-sensor co-movement patterns via cross-channel relational self-attention. The two orthogonal embeddings $z_{\mathrm{temp}}$ and $z_{\mathrm{sens}}$ are concatenated and passed through a fusion Multi-Layer Perceptron (MLP) that learns independent weights for each representational axis, producing a unified fused embedding $z=[z_{\mathrm{temp}}\;∥\;z_{\mathrm{sens}}]$ that carries complementary discriminative information unavailable to any single-stream architecture. Second, an Out-of-Distribution Detector for Neural Networks (ODIN) [39] gate, which monitors every incoming streaming [40] sample and autonomously triggers few-shot fault registration when a novel fault signature is detected, eliminating the need for any human-defined task boundary signal and directly addressing the OpenWorld problem. Third, a dual-space prototype bank combined with manifold mixup augmentation, which enables robust new fault class registration from as few as K∈{1,5,10} labeled support samples, addressing the class imbalance from few-shot constraints inherent to petrochemical fault data.

### 4.1. Offline Base Training: Dual Vision Transformer Classifier

**Figure 2:**
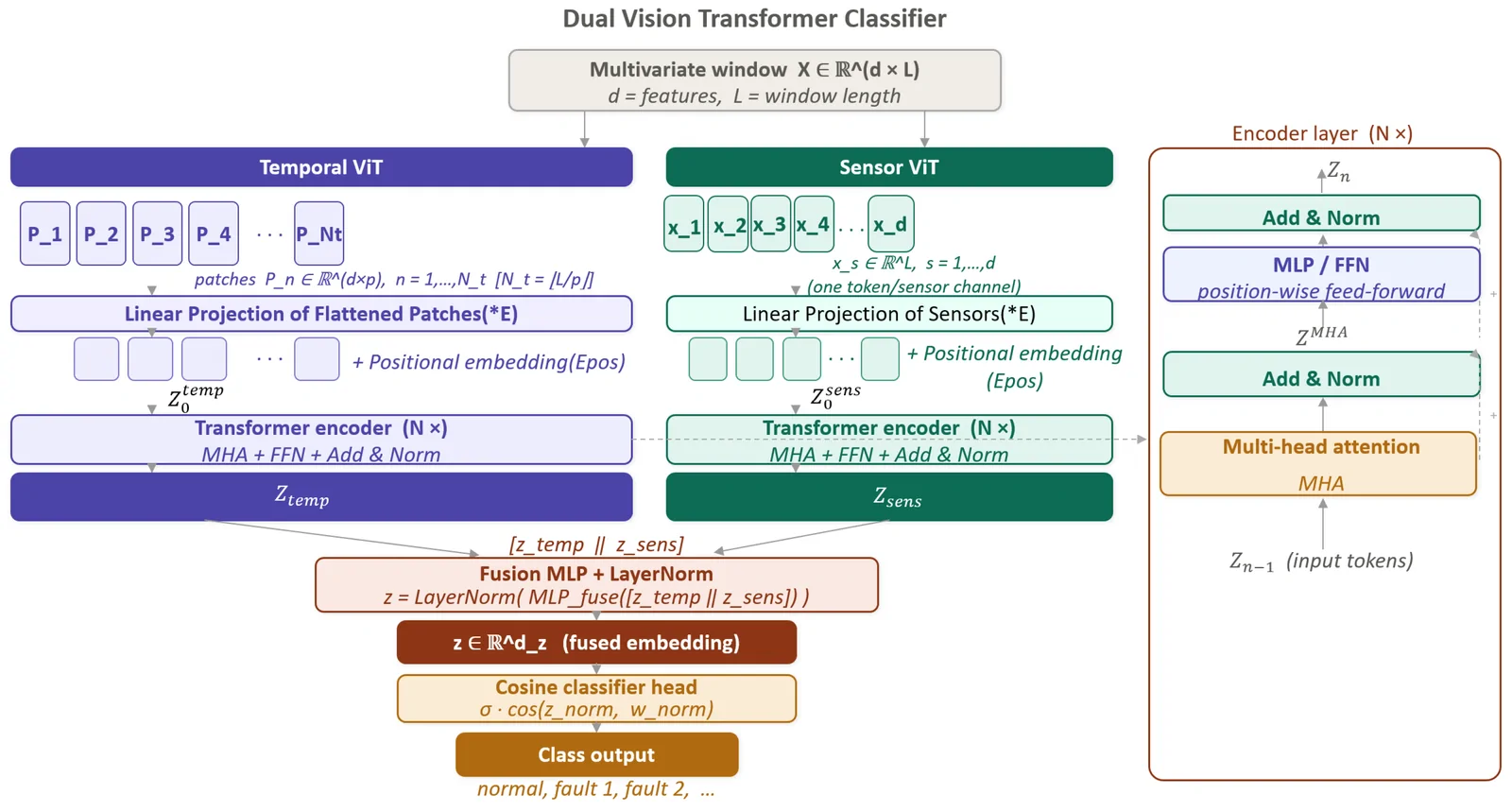
Structure of the Dual Vision Transformer.

The central architectural contribution of the proposed framework, as shown in Figure 2, is the dual-attention stages by two parallel Vision Transformers (ViTs)  [36], each processing the multivariate window from a semantically distinct axis. The two streams operate simultaneously, and their outputs are fused before any downstream classification or continual learning operation. This is depicted in Algorithm 1.

**Algorithm 1** OD-ViTFScL Base Learning Phase.**Require:** $D_{0}={\left\{\left(X_{i},y_{i}\right)\right\}}_{i=1}^{N}$, $C_{0}$, patch size *p*, *L*, $d_{\mathrm{model}}$, η, *E*, $N_{\mathrm{ewc}}$, *M* **Ensure:** $\theta^{*}$, $\Omega^{\mathrm{DR}}$, $M_{\mathrm{proto}}$, $M_{\mathrm{replay}}$  1:// Initialise model  2:Init Temporal ViT: $N_{t}\;=\;\left\lfloor L/p\right\rfloor$ patches  3:Init Sensor ViT: d=52 tokens  4:Init Fusion MLP and CosineClassifier($|C_{0}|$, σ)  5:$M_{\mathrm{replay}}\leftarrow \emptyset$  6:// Offline base training  7:**for** e=1 to *E* **do**  8:     **for** each batch $\left(X_{b},y_{b}\right)∼D_{0}$ **do**  9:           $z\leftarrow \mathrm{LayerNorm}({\mathrm{MLP}}_{\mathrm{fuse}}\left(\left[\mathrm{TViT}\left(X_{b}\right)∥\mathrm{SViT}\left(X_{b}\right)\right]\right))$10:           $L_{\mathrm{CE}}\leftarrow -\frac{1}{\left|B\right|}\sum_{i}\log p_{\theta }\left(y_{i}|z_{i}\right)$11:           Update θ via AdamW + cosine LR12:     **end for**13:**end for**14:$\theta^{*}\leftarrow \theta$15:// Populate replay buffer16:**for** each $\left(X_{i},y_{i}\right)\in D_{0}$ **do**17:      $M_{\mathrm{replay}}\left[y_{i}\right]\leftarrow M_{\mathrm{replay}}\left[y_{i}\right]\cup \left\{X_{i}\right\}$18:**end for**19:// Compute EWC-DR importance matrix20:Sample $B_{\mathrm{ewc}}∼M_{\mathrm{replay}}$, $|B_{\mathrm{ewc}}|=N_{\mathrm{ewc}}$21:**for** each batch $X_{b}\in B_{\mathrm{ewc}}$ **do**22:      ${\tilde{g}}_{k}\leftarrow -g_{k}$;   ${\tilde{p}}_{k}\leftarrow \exp\left({\tilde{g}}_{k}\right)/\sum_{j}\exp\left({\tilde{g}}_{j}\right)$23:      ${\tilde{L}}_{\mathrm{CE}}\leftarrow -\sum_{k}y_{k}\log{\tilde{p}}_{k}$24:      $\Omega^{\mathrm{DR}}\leftarrow \Omega^{\mathrm{DR}}+{\left(\nabla_{\theta }{\tilde{L}}_{\mathrm{CE}}\right)}^{2}$25:**end for**26:$\Omega^{\mathrm{DR}}\leftarrow \Omega^{\mathrm{DR}}/\left|B_{\mathrm{ewc}}\right|$27:// Build prototype bank28:**for** each $c\in C_{0}$ **do**29:      $\mu_{c}^{\mathrm{temp}},\;\mu_{c}^{\mathrm{sens}},\;\mu_{c}\leftarrow \frac{1}{|M_{c}|}\sum_{X\in M_{c}}z_{\mathrm{temp}},\;z_{\mathrm{sens}},\;z$30:      $M_{\mathrm{proto}}\leftarrow M_{\mathrm{proto}}\cup \left\{\mu_{c}^{\mathrm{temp}},\;\mu_{c}^{\mathrm{sens}},\;\mu_{c}\right\}$31:**end for**32:**return** $\theta^{*},\;\Omega^{\mathrm{DR}},\;M_{\mathrm{proto}},\;M_{\mathrm{replay}}$

#### 4.1.1. Temporal ViT: Patch-Based Temporal Self-Attention

When using the ViT architecture in the time-series domain to capture the temporal information from the process data, ViT treats the input multivariate window $X\in R^{d\times L}$ as a structured sequence by partitioning the time axis into $N_{t}=\left\lfloor L/p\right\rfloor$ non-overlapping patches of length *p*. *L* and *d* are the data timestamped window and number of features, respectively. Each patch $P_{n}\in R^{d\times p}$ is flattened and linearly projected with a learnable parameter *E*. This projection generates reduced dimensional tokens for each patch. Sinusoidal positional embeddings $E_{pos}$ are added to the flattened projected tokens to preserve the temporal ordering of the input data streams. The resulting token sequence, as represented in Equation (1), serves as the input for the Transformer encoder.

(1) $$z_{0}^{\mathrm{temp}}=E\cdot \mathrm{vec}\left(P_{n}\right)+E_{pos},\;n=1,\ldots ,N_{t}$$

The Transformer encoder shown in Figure 2 comprises *N* layers of multi-head attention (MHA) and feed-forward network (FFN) with residual connections and layer normalization. The MHA component allows the model to attend to different parts of the input data, while the FFN is a fully connected neural network  [36]. Equation (2), shows the temporal Transformer encoder multi-head attention output $z_{\mathrm{temp}}^{MHA}$, and Equation (3) shows the encoder output. This output is concatenated with the output from the sensor Transformer encoder $z_{\mathrm{sens}}$.

(2) $$z_{\mathrm{temp}}^{MHA}=\mathrm{LayerNorm}\;\left(\mathrm{MHA}\left(z_{n-1}\right)+z_{n-1}\right)$$

(3) $$z_{\mathrm{temp}}=\mathrm{LayerNorm}\;\left(\mathrm{FFN}\left(z_{\mathrm{temp}}^{MHA}\right)+z_{\mathrm{temp}}^{MHA}\right)$$

#### 4.1.2. Sensor ViT: Cross-Channel Relational Self-Attention

The Sensor ViT transposes the representational axis: rather than treating temporal patches as tokens, it treats each sensor channel s∈{1,…,d} as an individual token. The full time-series record of sensor *s* within the window is projected to a token embedding:

(4) $$z_{0}^{\mathrm{sens}}=E\cdot x_{s}+E_{pos},\;s=1,\ldots ,d$$

where $x_{s}\in R^{L}$ is the raw time series of sensor *s*. Learnable sensor-type positional embeddings $E_{pos}$, initialized by process variable group (flow, temperature, pressure, composition), provide structural priors that encode domain knowledge and accelerate convergence on new fault classes with limited support. The sensor tokens are processed through $L_{S}$ Transformer encoder layers as inputs and generate output embedding $z_{\mathrm{sens}}$ with an equation similar to (2). The self-attention mechanism of the Sensor ViT captures explicit cross-sensor correlations that are diagnostic of specific fault modes. For example, TEP Fault 2 (B-component feed loss) manifests as a structured co-movement between feed flow and reactor temperature sensors. The output $z_{\mathrm{sens}}$ from the final token constitutes the sensor-relational embedding. The sensor-type positional embedding is done on a grouping basis, so it can actually also be termed group embedding, with each group representing what each sensor measures. For example, in the TEP dataset, we have four groups for flow, temperature, pressure, and composition. Each sensor is assigned a group based on the physical category to which it belongs. Then, a fully learnable embedding matrix is created for each group; the values in the embedding matrix are randomly initialized. Hence, whenever new data is encountered, the group index for each sensor is used to lookup the corresponding row in the earlier created matrices. This is then added to the linear projection of the time-series sensor. Invariably, sensors in the same group receive the same positional embedding vector.

#### 4.1.3. Dual-Stream Fusion

The two embeddings from earlier sections are fused via concatenation followed by a two-layer fusion MLP with layer normalization:

(5) $$z=[z_{\mathrm{temp}}\;∥\;z_{\mathrm{sens}}]$$

Concatenation is used rather than additive fusion because the two streams carry orthogonal information—temporal dynamics versus cross-sensor topology—and their relative contributions should be independently weighted by the fusion network. The fused embedding *z* is the unified representation used for all downstream operations: classification, OOD detection, and continual learning.

#### 4.1.4. Classification

The fused embeddings from Equation (5) are fed into the decoder’s MLP to produce $z_{out}$, as shown in Equation (6). A cosine classifier for normalization and softmax probability calculation for fault class prediction, as shown in Equations (7) and  (8), are then applied. The cosine classifier eliminates the weight magnitude bias of the softmax classifier by normalizing both the embeddings vector $w_{c}$ and the class weight *z* vector to the unit sphere before computing the dot product, producing scores $g_{c}$, as seen in Equation (7), that depend purely on the angle between them rather than their magnitudes [41]; σ is a learnable scalar. This angular decision boundary ensures that newly imprinted few-shot class neurons, initialized directly from prototype embeddings at the unit norm, compete on equal geometric footing with existing classes from the first inference step, without being penalized for their smaller weight magnitudes. The cosine classifier is used at the output of the final layer to calculate the logits for classification.

(6) $$z_{\mathrm{out}}={\mathrm{MLP}}_{\mathrm{classifier}}\;\left(z\right)$$

(7) $$g_{c}=\sigma \cdot \frac{w_{c}}{∥w_{c}{∥}_{2}}\cdot \frac{z_{\mathrm{out}}}{∥z_{\mathrm{out}}{∥}_{2}}=\sigma \cdot \cos\left(w_{c},\;z_{\mathrm{out}}\right)$$

(8) $${\hat{y}}_{\mathrm{class}}=\arg\max_{c}\;g_{c}$$

### 4.2. Few-Shot Continual Learning

Our proposed method introduces the continual learning framework in two phases during the asynchronous streaming phase: Firstly, the arrival of new data is triggered by the ODIN method, which classifies it as being in-distribution (ID) data or out-of-distribution (OOD) data. The second phase is the update of the pre-trained model using few-shot and mixed augmented data. This is depicted in Algorithm 2.

**Algorithm 2** OD-ViTFScL Continual Learning—Streaming Phase.**Require:** Trained parameters $\theta^{*}$, importance matrix $\Omega^{\mathrm{DR}}$, prototype bank $M_{\mathrm{proto}}$, replay buffer $M_{\mathrm{replay}}$, accumulated classes $C_{k-1}$, streaming data ${\left\{\left(X_{t},y_{t}\right)\right\}}_{t=1}^{\infty }$, OOD threshold $\delta_{\mathrm{OOD}}$, OOD patience ρ, shot size *K*, EWC strength $\lambda_{\mathrm{EWC}}$, distillation strength $\lambda_{\mathrm{KD}}$, distillation temperature $T_{d}$, decay factor γ, mixup coefficient $\alpha_{\mathrm{mix}}$ **Ensure**: Updated θ, $\Omega^{\mathrm{DR}}$, $M_{\mathrm{proto}}$, $M_{\mathrm{replay}}$  1:Initialise OOD streak counter streak[c]←0 for all *c*  2:Initialise unknown buffer $B_{\mathrm{unk}}\leftarrow \emptyset$  3:**while** stream has next sample $\left(X_{t},y_{t}\right)$ **do**  4:     // — Feature extraction —  5:     $z_{\mathrm{temp}}\leftarrow \mathrm{TemporalViT}\left(X_{t}\right)$  6:     $z_{\mathrm{sens}}\leftarrow \mathrm{SensorViT}\left(X_{t}\right)$  7:     $z\leftarrow \mathrm{LayerNorm}({\mathrm{MLP}}_{\mathrm{fuse}}\left(\left[z_{\mathrm{temp}}∥z_{\mathrm{sens}}\right]\right))$  8:     // — Phase II: ODIN OOD detection —  9:     ${\tilde{X}}_{t}\leftarrow X_{t}-\epsilon \cdot \mathrm{sign}\left(-\nabla_{X}\log\max_{c}{\hat{p}}_{t,c}\right)$10:     $S\left(X_{t}\right)\leftarrow \max_{c}{\hat{p}}_{t,c}\left({\tilde{X}}_{t}/T\right)$11:     $D_{\mathrm{proto}}\leftarrow \min_{c\in C_{k-1}}{∥z-\mu_{c}∥}_{2}$12:     **if** $S\left(X_{t}\right)>\delta_{\mathrm{OOD}}$ and $D_{\mathrm{proto}}<\tau$ **then**13:         // — Known class: update model —14:         ${\hat{y}}_{t}\leftarrow \arg\max_{c}\;g_{c}$15:         Build mixed batch: $B\leftarrow \left\{\left(X_{t},{\hat{y}}_{t}\right)\right\}\cup R_{k-1}$16:         Compute CE loss: $L_{\mathrm{CE}}\leftarrow -\frac{1}{\left|B\right|}\sum_{(X_{i},y_{i})\in B}\log p_{\theta }\left(y_{i}|z_{i}\right)$17:         Compute EWC-DR penalty: $L_{\mathrm{EWC}}\leftarrow \lambda_{\mathrm{EWC}}\sum_{i}\Omega_{i}^{\mathrm{DR}}{\left(\theta_{i}-\theta_{i}^{*}\right)}^{2}$18:         Compute KD loss: $L_{\mathrm{KD}}\leftarrow -\lambda_{\mathrm{KD}}\sum_{c\in C_{k-1}}p_{\theta^{*}}\left(c|z\right)\log p_{\theta }\left(c|z\right)$19:         $L_{\mathrm{total}}\leftarrow L_{\mathrm{CE}}+L_{\mathrm{EWC}}+L_{\mathrm{KD}}$20:         Update θ via Adam, $\nabla_{\theta }L_{\mathrm{total}}$21:         Push $X_{t}$ to $M_{\mathrm{replay}}\left[{\hat{y}}_{t}\right]$22:     **else**23:           // — Unknown class: buffer and detect —24:           $B_{\mathrm{unk}}\left[y_{t}\right]\leftarrow B_{\mathrm{unk}}\left[y_{t}\right]\cup \left\{X_{t}\right\}$25:           $\mathrm{streak}\left[y_{t}\right]\leftarrow \mathrm{streak}\left[y_{t}\right]+1$26:           **if** $\mathrm{streak}[y_{t}]\geq \rho$ **then**27:               // — Phase III: Few-shot registration —28:               $c_{\mathrm{new}}\leftarrow y_{t}$,   $C_{k}\leftarrow C_{k-1}\cup \left\{c_{\mathrm{new}}\right\}$29:               Collect *K* support samples: $S_{c_{\mathrm{new}}}\leftarrow B_{\mathrm{unk}}{\left[c_{\mathrm{new}}\right]}_{1:K}$30:               *Step 1—Dual-space prototype construction:*31:               $\mu_{c_{\mathrm{new}}}^{\mathrm{temp}}\leftarrow \frac{1}{K}\sum_{i=1}^{K}z_{\mathrm{temp}}^{\left(i\right)}$32:               $\mu_{c_{\mathrm{new}}}^{\mathrm{sens}}\leftarrow \frac{1}{K}\sum_{i=1}^{K}z_{\mathrm{sens}}^{\left(i\right)}$33:               $\mu_{c_{\mathrm{new}}}\leftarrow \frac{1}{K}\sum_{i=1}^{K}z^{\left(i\right)}$34:               $M_{\mathrm{proto}}\leftarrow M_{\mathrm{proto}}\cup \left\{\mu_{c_{\mathrm{new}}}^{\mathrm{temp}},\;\mu_{c_{\mathrm{new}}}^{\mathrm{sens}},\;\mu_{c_{\mathrm{new}}}\right\}$35:               *Step 2—Manifold mixup augmentation:*36:               $c_{\mathrm{nn}}\leftarrow \arg\min_{c\neq c_{\mathrm{new}}}∥\mu_{c}-\mu_{c_{\mathrm{new}}}∥$37:               $\lambda_{1}∼\mathrm{Beta}\left(\alpha_{\mathrm{mix}},\alpha_{\mathrm{mix}}\right)$,   $\lambda_{1}\leftarrow \max\left(\alpha_{\mathrm{mix}},\lambda_{1}\right)$38:               ${\tilde{z}}_{\mathrm{aug}}\leftarrow \lambda_{1}\mu_{c_{\mathrm{new}}}+\left(1-\lambda_{1}\right)\mu_{c_{\mathrm{nn}}}$39:               *Step 3—Replay buffer update:*40:               Push $S_{c_{\mathrm{new}}}$ to $M_{\mathrm{replay}}\left[c_{\mathrm{new}}\right]$41:               // — Phase IV: Incremental update —42:               Snapshot teacher: $\theta^{\left(k-1\right)}\leftarrow \theta$43:               Expand classifier head by 1 neuron44:               Imprint weight: $w_{c_{\mathrm{new}}}\leftarrow \mu_{c_{\mathrm{new}}}/∥\mu_{c_{\mathrm{new}}}∥$45:               Build mixed batch: $B\leftarrow S_{c_{\mathrm{new}}}\cup \left\{{\tilde{z}}_{\mathrm{aug}}\right\}\cup R_{k-1}$46:               Compute $L_{\mathrm{total}}=L_{\mathrm{CE}}+L_{\mathrm{EWC}}+L_{\mathrm{KD}}$ and update θ47:               *Online EWC-DR update:*48:               Sample $B_{\mathrm{ewc}}∼M_{\mathrm{replay}}$49:               $\Omega^{\mathrm{DR},(k)}\leftarrow E\;\left[{\left(\nabla_{\theta }{\tilde{L}}_{\mathrm{CE}}\right)}^{2}\right]$ on $B_{\mathrm{ewc}}$ via logits reversal50:               ${\hat{\Omega }}^{\left(k\right)}\leftarrow \gamma \;{\hat{\Omega }}^{\left(k-1\right)}+\left(1-\gamma \right)\;\Omega^{\mathrm{DR},(k)}$51:               *Prototype refresh:*52:               **for** each class $c\in C_{k}$ **do**53:                   $\mu_{c}\leftarrow \frac{1}{|M_{c}|}\sum_{X\in M_{c}}f_{\theta }\left(X\right)$54:               **end for**55:               $C_{k-1}\leftarrow C_{k}$,   $\theta^{*}\leftarrow \theta$,   $\mathrm{streak}[y_{t}]\leftarrow 0$56:           **end if**57:     **end if**58:**end while**59:**return** $\theta ,\;\Omega^{\mathrm{DR}},\;M_{\mathrm{proto}},\;M_{\mathrm{replay}}$

#### 4.2.1. Phase I: Out-of-Distribution Detection via ODIN

During the asynchronous streaming phase, each incoming sample $X_{t}$ passes through the full DViT extraction pipeline to produce a fused embedding *z* and a logit vector $g_{t}$. Before any classification decision is made, an OOD detection gate is applied using the ODIN framework to trigger the arrival of new data. The ODIN technique involves two approaches, namely, temperature scaling and input perturbation.

Temperature-scaled softmax, shown in Equation (9), is calculated using the pre-trained model output logit $g_{t,c}$ and a temperature scaling parameter T. The temperature scaling aids in distinct separation between the ID and OOD confidence scores.

(9) $${\hat{p}}_{t,c}=\frac{\exp(g_{t,c}/T)}{\sum_{c^{\prime }}\exp\left(g_{t,c^{\prime }}/T\right)}$$

Input perturbation, as shown in Equation (10), is applied to the input data before the temperature scaling to nudge the input in a direction that increases probability for the anticipated class—ID or OOD; ϵ is the perturbation magnitude.

(10) $${\tilde{X}}_{t}=X_{t}-\epsilon \cdot \mathrm{sign}\;\left(-\nabla_{X}\log\max_{c}{\hat{p}}_{t,c}\right)$$

The final detection rule is

(11) $$\mathrm{Decision}\left(X_{t}\right)=\left\{\begin{array}{cc} \mathrm{ID}\;\to \;{\hat{y}}_{t}=\arg\max_{c}{\hat{p}}_{t,c}\; & \mathrm{if}\;\max_{c}{\hat{p}}_{t,c}\left({\tilde{X}}_{t}\right)>\delta_{\mathrm{OOD}}\\ \mathrm{OOD}\;\to \;\mathrm{trigger}\;\mathrm{few}\text{-}\mathrm{shot}\;\mathrm{registration}\; & \mathrm{otherwise} \end{array}\right.$$

If the maximum of the probability shown in Equation (9) is greater than declared OOD threshold $\delta_{\mathrm{OOD}}$, the data is ID; otherwise, it is OOD.

#### 4.2.2. Phase II: Few-Shot Class Registration

Few-shot class registration occurs when the OOD gate triggers; the system accumulates *K* labeled support samples $S_{c_{\mathrm{new}}}={\left\{\left(X_{i},c_{\mathrm{new}}\right)\right\}}_{i=1}^{K}$ and registers the new fault through three coordinated steps. The K labeled support samples are expert hand-labeled samples that are acquired immediately after ODIN triggers the arrival of new OOD data. Our ODIN application here is to trigger the arrival of novel classes by observing the out-of-distribution dataset, not labeling of the samples.

Step 1—Dual-Space Prototype Construction:
  (12) $$\mu_{c_{\mathrm{new}}}^{\mathrm{temp}}=\frac{1}{K}\sum_{i=1}^{K}z_{\mathrm{temp}}^{\left(i\right)},\;\mu_{c_{\mathrm{new}}}^{\mathrm{sens}}=\frac{1}{K}\sum_{i=1}^{K}z_{\mathrm{sens}}^{\left(i\right)},\;\mu_{c_{\mathrm{new}}}=\frac{1}{K}\sum_{i=1}^{K}z^{\left(i\right)}$$
Step 2—Manifold Mixup Augmentation: Generates augmented embeddings from the K support samples prototype and the nearest existing prototype to the new class:

(13) $${\tilde{z}}_{\mathrm{aug}}=\lambda_{1}\mu_{c_{\mathrm{new}}}+\left(1-\lambda_{1}\right)\mu_{c_{\mathrm{nn}}},\;$$

where $\mu_{c_{\mathrm{nn}}}=\arg\min_{c\neq c_{\mathrm{new}}}∥\mu_{c}-\mu_{c_{\mathrm{new}}}∥$ is the nearest existing prototype and $\lambda_{1}$ is not a fixed value but is sampled from a $\mathrm{Beta}(\alpha_{\mathrm{mix}},\alpha_{\mathrm{mix}})$ distribution.

- $\alpha_{\mathrm{mix}}<1$ (e.g., 0.2): The distribution is U-shaped, concentrating mass near 0 and 1, so $\lambda_{1}$ typically falls close to one prototype or the other, yielding gentle interpolation. This is the value selected for our experiment.
- $\alpha_{\mathrm{mix}}=1$: The distribution is uniform on [0,1], making every mixing ratio equally likely.
- $\alpha_{\mathrm{mix}}>1$: The distribution is bell-shaped, concentrating mass near 0.5, which produces aggressive blending at the midpoint between the two prototypes.

The augmented embeddings mixup generate synthetic data from the new k samples and existing classes for downstream training of the model. This augmentation occurs in the fused embedding space, which utilizes the proposed Dual ViT framework.

Step 3—Replay Buffer Update: A reservoir-sampled subset of the new class support embeddings is added to $M_{\mathrm{replay}}$, maintaining balanced representation across all accumulated classes $C_{k}$. The replay buffer stores raw samples.

#### 4.2.3. Phase III: Model Update

Cross-entropy loss $L_{\mathrm{CE}}$ is computed over a mixed mini-batch B comprising the *K* few-shot support samples for the new class, their manifold mixup-augmented counterparts, and a proportional sample of replay exemplars drawn from all prior classes in $M_{\mathrm{replay}}$. This loss enables the model to classify the data accurately from the purity of the mixed mini-batch data.

(14) $$L_{\mathrm{CE}}=-\frac{1}{\left|B\right|}\sum_{(X_{i},\;y_{i})\;\in \;B}\sum_{c=1}^{|C_{k}|}\left[y_{i}=c\right]\;\log p_{\theta }\left(c|z_{i}\right)$$

where $p_{\theta }\left(c|z_{i}\right)$ is the predicted probability for class *c* produced by the cosine classifier head for the fused embedding $z_{i}$, $C_{k}=C_{k-1}\cup \left\{c_{\mathrm{new}}\right\}$ is the accumulated class set at task $T_{k}$, and the mixed mini-batch is defined as follows:

(15) $$B=S_{c_{\mathrm{new}}}\;\cup \;S_{c_{\mathrm{new}}}^{\mathrm{aug}}\;\cup \;R_{k-1}$$

with $S_{c_{\mathrm{new}}}={\left\{\left(X_{i},c_{\mathrm{new}}\right)\right\}}_{i=1}^{K}$ denoting the *K* few-shot support samples, $S_{c_{\mathrm{new}}}^{\mathrm{aug}}$ the manifold mixup-augmented embeddings from Equation (13), and $R_{k-1}$ a proportional sample of replay exemplars drawn from $M_{\mathrm{replay}}$ across all prior classes $C_{k-1}$.

Elastic Weight Consolidation Done Right (EWC-DR) [42] loss is utilized for eliminating vanishing gradients in vanilla EWC, where weight importance is estimated via the diagonal Fisher Information Matrix (FIM). To compute the FIM as seen in Equation (17), each logit $g_{k}$ is negated to ${\tilde{g}}_{k}=-g_{k}$ before the softmax, so that high-confidence correct predictions produce large gradients rather than vanishing ones. The reversed cross-entropy loss is then differentiated with respect to every trainable parameter in the network, and the squared gradients are accumulated over a batch of replay buffer samples. The computation is performed in mini-batches of samples drawn from the replay buffer, with gradients accumulated and averaged across all batches to produce the final importance matrix $\Omega^{\mathrm{DR}}$. This continual learning technique penalizes weights that are important to previous tasks to mitigate the catastrophic forgetting problem.

(16) $$L_{\mathrm{EWC}}=\sum_{i}\Omega_{i}^{\mathrm{DR}}\;{\left(\theta_{i}-\theta_{i}^{*}\right)}^{\;2}$$

(17) $$\Omega_{i}^{\mathrm{DR}}=E\;\left[{\left(y_{k}-{\tilde{p}}_{k}\right)}^{\;2}\cdot {\left(\frac{\partial {\tilde{g}}_{k}}{\partial \theta_{i}}\right)}^{\;2}\right]$$

The FIM is updated at convergence of base learning, at every novel class registration, and during online learning. It uses raw data in the replay buffer rather than embeddings, and it applies to all layers, as it is computed by backpropagation through the entire network using the chain rule.

Knowledge distillation loss that transfers knowledge from the pre-trained ViT to the newly updated model is as shown in Equation (18):

(18) $$L_{\mathrm{KD}}=-\sum_{c\in C_{k-1}}p_{\theta^{\left(k-1\right)}}\left(c|z\right)\;\log p_{\theta^{\left(k\right)}}\left(c|z\right)$$

where the temperature-scaled probability is

(19) $$p_{\theta }\left(c|z\right)=\frac{\exp\;\left(g_{c}\;/\;T_{d}\right)}{\sum_{c^{\prime }}\exp\;\left(g_{c^{\prime }}\;/\;T_{d}\right)}$$

where $T_{d}$ is the temperature scalar that determines how much of the old model’s knowledge is transferred to the newly updated model.

The composite loss function is

(20) $$L_{\mathrm{total}}=L_{\mathrm{CE}}+\lambda_{\mathrm{EWC}}L_{\mathrm{EWC}}+\lambda_{\mathrm{KD}}\;L_{\mathrm{KD}}$$

For the validity of the embeddings after a model update, two techniques help with this: the Elastic Weight Consolidation, and the knowledge distillation (KD) loss. The EWC-DR regularization term directly limits how much the backbone parameters can move during each incremental update. Parameters with high importance, i.e., those most responsible for producing discriminative embeddings for known fault classes, are strongly penalized from moving. The knowledge distillation loss computed by passing replay-buffer raw inputs through both the teacher and the student penalizes any change in the output probability distribution over old classes, which implicitly constrains how much the embedding can shift for old-class inputs. If the embedding drifted significantly, the cosine classifier logits would change, producing a large KD loss that forces the update to preserve the functional mapping from inputs to class probabilities for all previously learned faults.

## 5. Experiments

### 5.1. Dataset Description

TEP: This dataset consists of 20 distinct fault types as well as one fault-free state, making it a 21-class dataset [43,44,45]. Each fault type and the fault-free state contain 500 simulations, giving a total of 10,500 simulations. Each simulation contains 25 h of operation with a sampling time of 3 min, providing 500 data samples per run. There are 52 features in this dataset: 41 process variables and 11 manipulated variables. For our experiments, we utilize Faults 1–9 and normal states in class-imbalanced settings: 500 normal and 48 individual fault class samples for training, and 800 samples for both normal and faulty classes in testing.

The window is extracted from a timestamped sequence of pre-built arrays produced by a function that reads from the raw simulation files stated above. The window length is 30, stride is 1, and a min–max normalization is applied.

The entire operating sequences from the dataset were separated out for training and testing to avoid temporal data leakage that could be attributed to overestimated model performance.

Scenario Setup:

(21) $$\left\{\begin{array}{cc} \mathrm{At}\;\mathrm{known}\;\mathrm{time}\;t_{0}, & T_{0}=\left\{0,1\right\}\\ \mathrm{At}\;\mathrm{unknown}\;\mathrm{time}\;t_{1}, & T_{1}=\left\{2,3\right\}\\ \mathrm{At}\;\mathrm{unknown}\;\mathrm{time}\;t_{2}, & T_{2}=\left\{4,5\right\}\\ \mathrm{At}\;\mathrm{unknown}\;\mathrm{time}\;t_{3}, & T_{3}=\left\{6,7\right\}\\ \mathrm{At}\;\mathrm{unknown}\;\mathrm{time}\;t_{4}, & T_{4}=\left\{8,9\right\} \end{array}\right.$$

where $T_{0}$ is the base learning phase and $T_{1}$–$T_{4}$ are incremental streaming sessions, arriving asynchronously such that

(22) $$t_{1}\neq t_{2}\neq t_{3}\neq t_{4}\;(\mathrm{Asynchronous}\;\mathrm{Streaming})$$

CWRU: This dataset consists of 9 distinct fault types and normal operation of a motor shaft bearing support located in one of three parts of the bearing: ball, inner race, and outer race. The total sample size is 1265. The scenario setup follows the same structure shown in Equation (21) and Table 1. Training uses 115 normal and 12 individual fault class samples, and testing uses 115 samples for both normal and faulty classes.

### 5.2. Model Settings

Hyperparameter Settings: The model was trained with the following hyperparameter configuration: During the base learning phase, the model was trained for 80 epochs with a learning rate of η=0.003. For the continual learning regularization terms, the EWC penalty weight was set to $\lambda_{\mathrm{EWC}}=0.2$ and the knowledge distillation weight to $\lambda_{\mathrm{KD}}=1.5$. Out-of-distribution detection via ODIN was configured with a confidence threshold of $\delta_{\mathrm{OOD}}=0.9$ and an input perturbation magnitude of ϵ=0.001; $\lambda_{1}$ in the beta prototype mixup was 0.4; the replay buffer was allocated a capacity of 400 embeddings, with 128 exemplars sampled per incremental step. For the Dual Vision Transformer backbone, the patch size was p=1, number of tokens $N_{t}=30$, dropout rate = 0.1, MLP hidden dimension = 512, the activation function was GELU, the optimizer was AdamW, the batch size for base training was 128 and 256 for streaming, the parameter count was 4,661,505, and the embedding dimension was set to $d_{\mathrm{model}}=256$, with N=4 Transformer encoder layers and H=4 attention heads in each multi-head self-attention block.

The ODIN threshold was initialized to 0.9 and empirically checked by plotting the max-softmax scores on held-out validation samples from the in-distribution dataset after base training and one novel sample to visualize the confidence scores’ separation. During streaming of new faults, at every 200 steps we computed the ODIN fire rate, representing the fraction of true novel samples flagged as OOD. If the rate falls below 5% with at least 32 novel samples observed, it bumps the threshold up by 0.02, capped at 0.995.

### 5.3. Evaluation Metrics

We evaluated the CL models using average accuracy (AA)—as shown in Equation (23)—which measures overall performance at a given moment, and backward transfer (BWT)—shown in Equation (24)—which is a memory stability metric to measure the impact of learning a new task on old tasks [7,46]:

(23) $$AA_{k}=\frac{1}{k}\sum_{j=1}^{k}a_{k,j}$$

(24) $${\mathrm{BWT}}_{k}=\frac{1}{k-1}\sum_{j=1}^{k-1}\left(a_{k,j}-a_{j,j}\right),$$

where $a_{k,j}$ is the accuracy estimated on the *j*-th task after incremental learning of the *k*-th task.

Baselines: For the purpose of fair comparison with our work, ODIN is used in the baselines to trigger the arrival of new class. DGGN [47] decouples feature learning into two parallel streams. iCARL [48] pioneers rehearsal-based incremental learning via exemplar memory and distillation. WaRP-CIFSL [49] consolidates knowledge by warping parameter space. SCFLID [50] tailors contrastive distillation for industrial incremental settings.

## 6. Results

**Figure 3:**
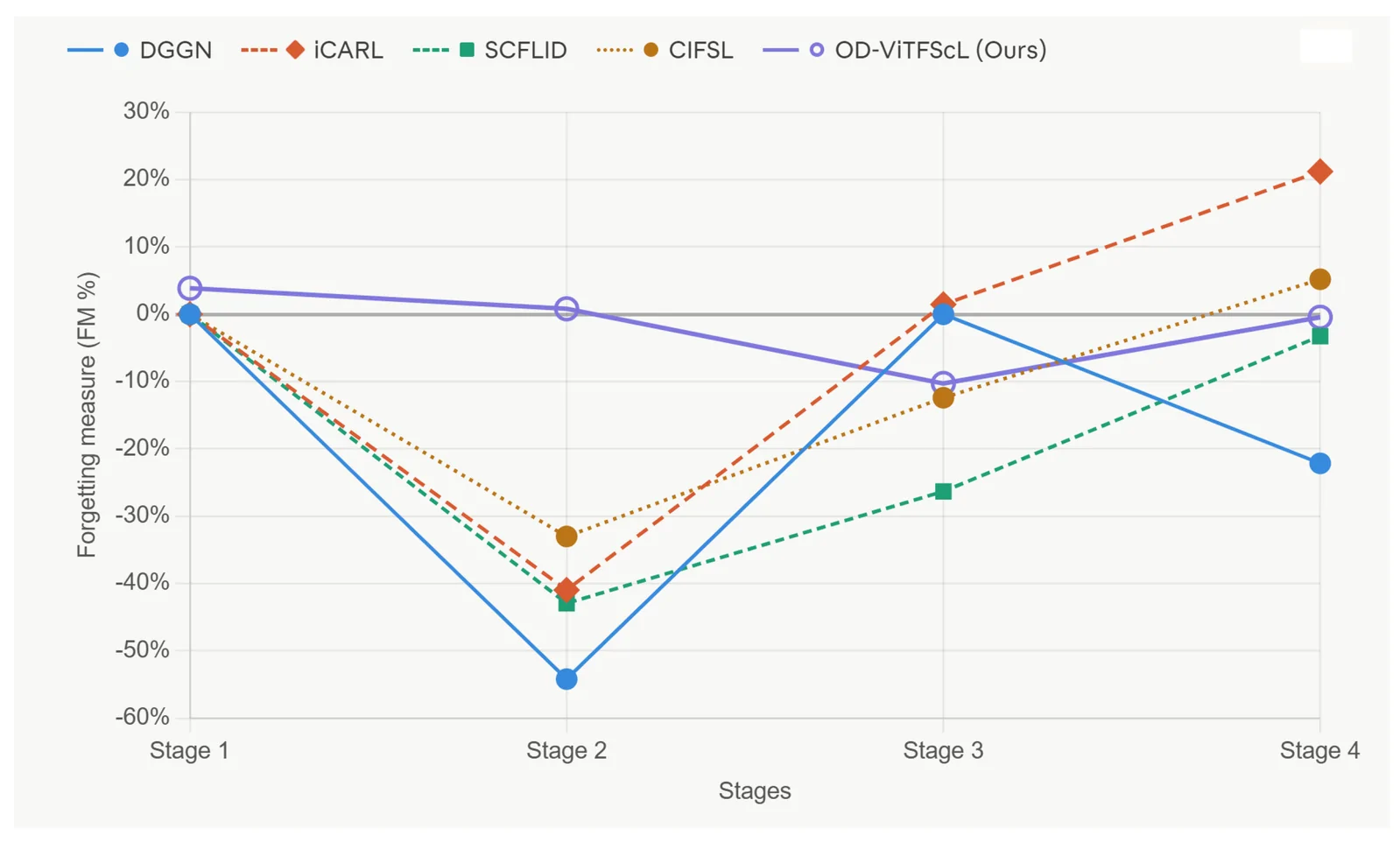
Our OD-ViTFScL memory stability curve compared to baselines.

The experimental results presented in Table 2 and Table 3 and Figure 3 collectively demonstrate that OD-ViTFScL consistently outperforms all baseline methods across average accuracy, backward transfer (BWT), precision, recall, and F1-score metrics on the TEP dataset.

To further validate the generalizability of the proposed framework, we conducted an additional experiment on the CWRU bearing fault dataset, reporting the per-stage and average accuracies.

As shown in Table 2, OD-ViTFScL achieved the highest average accuracy of 71.84%, representing an improvement of 19.36% over the next best-performing baselines iCARL and SCFLID (both 52.48%), and a 35.55% improvement over CIFSL (36.29%). This performance advantage was consistent and substantial across individual incremental sessions. Most notably, OD-ViTFScL achieved 99.00% accuracy on $T_{2}$ ([4, 5]), which represents near-perfect classification of classes introduced at the second incremental session—a result that no baseline approaches, with the closest competitor iCARL reaching only 77.69% on the same session.

A consistent pattern observed across all baselines is a severe accuracy collapse at $T_{3}$ ([6, 7]), where DGGN drops to 19.56%, iCARL to 15.94%, and CIFSL to 13.06%. This collapse is characteristic of catastrophic forgetting compounded by class imbalance in few-shot settings: when the model is forced to register two new fault classes with minimal support while retaining five previously learned classes, the decision boundaries of older classes are disrupted. OD-ViTFScL is the only method that maintains meaningful accuracy at $T_{3}$ (51.69%), attributable to the joint effect of EWC-DR regularization anchoring critical parameters in weight space and knowledge distillation preserving the output distribution of previously learned fault classes.

Further analysis is shown in Table 4 for the accuracy results reported in Table 2. The 5-fold cross validation runs on our method OD-VitFScL gave mean accuracy of 73.75%, validating our model’s high performance. We further performed a statistical *t*-test on our framework with the next-best baseline, SCFLID. The *t*-test result gave a *t* value of 2.435 with a *p*-value of 0.04, indicating statistical significance at the 5% level. We can conclude that the accuracy difference between the two models is unlikely to be due to chance, and that our model is genuinely better than the others.

Table 3 provides a more granular view of classification quality across incremental stages. A key observation is that all methods, including OD-ViTFScL, exhibit reduced macro precision and recall at Stage 0 relative to later stages. This is expected: at Stage 0, the model is exposed to only two classes ([0, 1]) under severe class imbalance (500 normal versus 48 fault samples), and macro-averaging equally weights both classes regardless of their support, penalizing performance on the minority fault class.

From Stage 1 onward, OD-ViTFScL demonstrates a decisive and sustained advantage. At Stage 1 ([0–3]), OD-ViTFScL achieves a macro precision of 72.19%, macro recall of 73.03%, and macro F1 of 67.66%—compared to CIFSL’s best baseline values of 17.77%, 35.53%, and 23.32%, respectively. This represents a macro F1 improvement of over 44 percentage points at Stage 1 alone, demonstrating that the Dual Vision Transformer backbone combined with the ODIN-triggered few-shot registration mechanism successfully integrates new fault classes into the classifier without degrading existing class boundaries.

At Stage 2 ([0–5]), OD-ViTFScL achieves the highest macro precision (74.29%) and macro F1 (63.26%) of any method at any stage, while maintaining competitive recall (69.08%). The progressive decline in macro F1 from Stage 1 (67.66%) to Stage 4 (70.57%) is non-monotonic, reflecting the dynamic interaction between new class registration and the replay-based consolidation mechanism. Notably, Stage 4 macro F1 (70.57%) recovers above the Stage 3 value (69.47%), suggesting that the online EWC-DR importance update and manifold mixup augmentation help re-stabilize decision boundaries as the cumulative class set grows.

CIFSL achieves the best macro precision at Stage 0 (76.06%) owing to its parameter-space warping strategy being particularly effective for binary classification, but it degrades rapidly in subsequent stages as the number of accumulated classes increases, falling to 32.66% macro precision at Stage 4. This pattern is consistent with the known limitation of parameter-warping approaches under extended incremental sequences: the warp capacity is finite and is exhausted as the class set grows beyond the method’s effective operating range.

**Figure 4:**
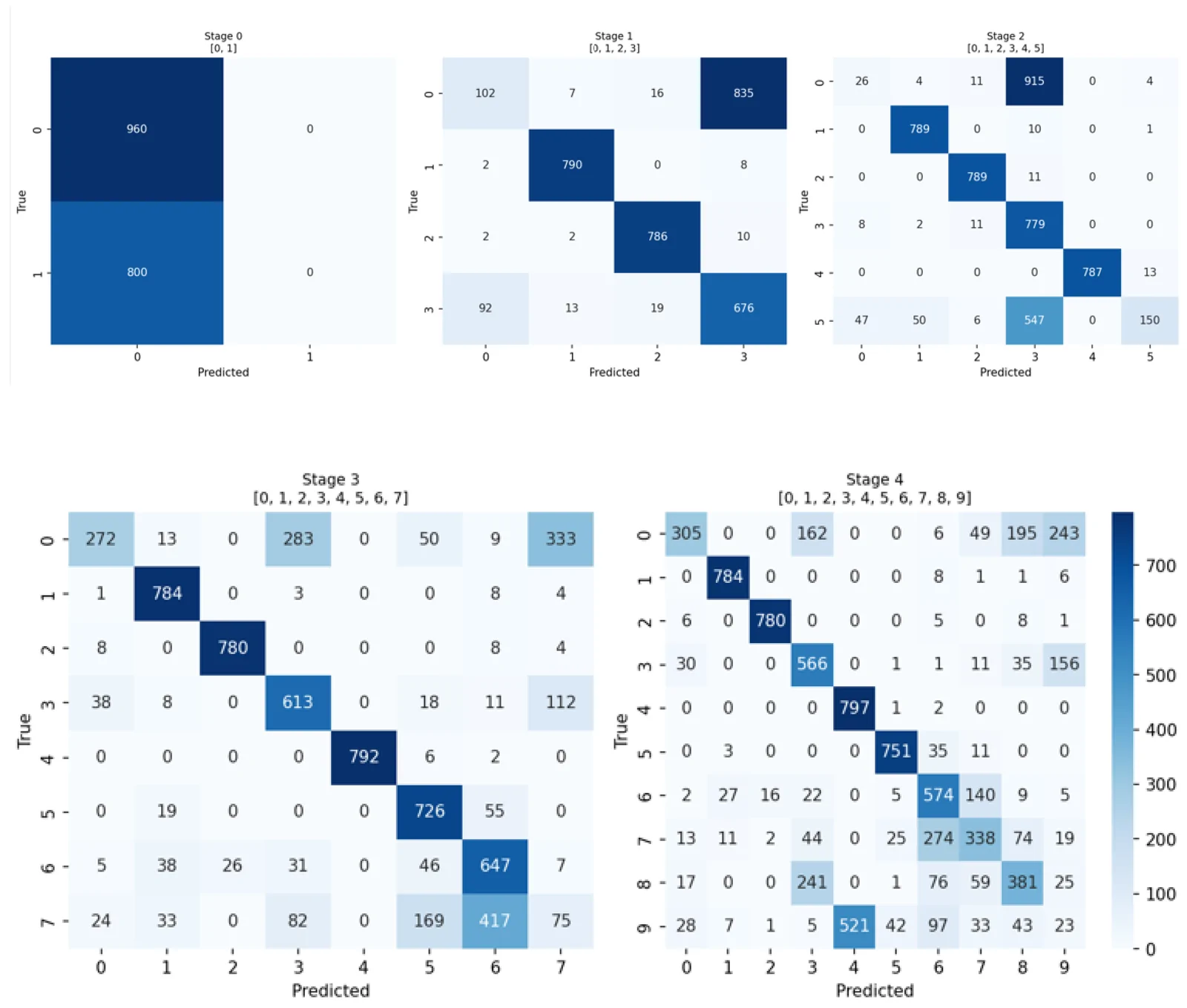
OD-VitFScL confusion matrix for TEP.

Across the five incremental stages, the confusion matrices shown in Figure 4 reveal a consistent pattern of strong base-class retention progressively challenged by inter-class interference as the class set expands. As shown in the matrix, at Stage 0, the model classifies all 1760 samples as class 0, yielding zero true positives for class 1 and collapsing its precision to 0.0. The class collapse and unbalanced datasets result in the proposed model’s poor performance at Stage 0. Stage 1 maintains strong performance on classes 1 and 2 (790 and 786 correct) but shows early signs of class 3 confusion, with 92 samples misclassified as class 0, indicating that the few-shot support for class 3 was insufficient to fully separate it from the base classes. Stage 2 introduces the most notable single-class failure in class 5, which records only 547 correct predictions alongside significant misclassification into classes 0, 1, and 5 itself, suggesting that fault class 5 shares overlapping temporal and cross-sensor signatures with earlier classes in the fused embedding space. Stage 3 reveals the most severe forgetting episode across the entire experiment, with class 0 deteriorating to only 272 correct predictions and 333 samples incorrectly assigned to class 7, while class 7 simultaneously absorbs heavy misclassification from classes 3, 5, and 6—a pattern consistent with the newly registered classes 6 and 7 encroaching on the prototype regions of previously well-separated base classes. Stage 4 shows a partial recovery for some classes—notably classes 1, 2, and 4, which maintain 784, 780, and 797 correct predictions, respectively—but class 0 continues to suffer persistent interference from classes 8 and 9 (195 and 243 misclassifications), while class 9 is the most confused class overall, with only 23 correct predictions, reflecting both the limited few-shot support available at its late registration and its structural similarity to classes 3, 4, 5, and 6 in the TEP process variable space. The macro F1-score of 69.48% at Stage 3 captures the cumulative effect of these failure modes, with class 7 achieving an F1 of only 0.1124 being the primary drag—a consequence of 417 of its 800 samples being absorbed by class 6, pointing to the need for stronger inter-class boundary enforcement between structurally proximate fault types registered in the same incremental task.

To verify the applicability of our method to faults arriving randomly in the incremental tasks, we further conducted experiments on two faults’ arrival forms, in addition to the earlier setup in the experiments on the TEP dataset. The experimental setup remained consistent with previous settings, and we evaluated the classification performance at each incremental stage throughout the entire incremental learning process. As shown in Table 5, under the class-imbalanced setting of the TEP dataset, the average classification accuracy for all groups exceeds 70.00%. These results further validate the robustness of our framework under different random fault arrival orders.

To validate the asynchronous hypothesis in our work, we report the performance metric for our ODIN method, which triggers the arrival of novel class distributions. The ODIN fire rate is 85.4%, with a false alarm rate of 33.3% and unregistered novel distribution of 0%. This indicates that our asynchronous streaming protocol achieved optimal performance.

Table 6 reveals the most striking distinction between OD-ViTFScL and all baseline methods. The ${\mathrm{BWT}}_{k}$ values of the baselines exhibit large-amplitude divergence across the incremental stages. DGGN reaches a minimum of −54.20% at Stage 2, indicating the most severe negative backward transfer among all methods—meaning that learning new fault classes substantially degrades performance on previously learned ones—before recovering to 0.00% at Stage 3 and declining again to −22.15% at Stage 4, yielding the lowest average ${\mathrm{BWT}}_{k}$ of −19.09%. iCARL presents a contrasting but equally problematic trajectory: after 0.00% at Stage 1, it records −40.96% at Stage 2, before reversing direction to +1.46% at Stage 3 and climbing to +21.21% at Stage 4, with an average ${\mathrm{BWT}}_{k}$ of −4.57%, indicating performance degradation on old fault classes as new ones are introduced. SCFLID and CIFSL follow similar patterns of large negative backward transfer at Stage 2 (−42.94% and −33.01%, respectively), with average ${\mathrm{BWT}}_{k}$ values of −18.13% and −10.05%, both reflecting substantial instability across the incremental sequence.

In contrast, OD-ViTFScL achieves the best average ${\mathrm{BWT}}_{k}$ of −1.51%, with stage-wise values of +3.86%, +0.81%, −10.28%, and −0.44% at Stages 1 through 4, respectively, demonstrating near-perfect backward transfer prevention throughout the full five-session incremental sequence. The piecewise linear nature of the memory stability curves shown in Figure 3 further highlights the instability of the baselines. For instance, DGGN’s abrupt −54.20% drop at Stage 2, followed by full recovery to 0.00% at Stage 3 and then a decline again to −22.15% at Stage 4, is characteristic of a method that lacks a principled mechanism for managing inter-task parameter interference, relying instead on coincidental gradient alignment. In contrast, OD-ViTFScL maintains near-zero lines across all four stages, indicating a superior memory stability performance.

### 6.1. CWRU Results

OD-ViTFScL achieves the highest average accuracy of 84.96% across all five incremental sessions on the CWRU bearing fault dataset, as shown in Table 7, outperforming the next-best baseline DGGN by 3.31 percentage points, and surpassing iCARL, SCFLID, and CIFSL by margins of 12.79, 4.17, and 5.83 percentage points, respectively. The proposed method attains the best per-task accuracy on the base session $T_{0}$ (85.65%), demonstrating that the offline base training phase establishes a strong discriminative foundation before streaming begins. Particularly notable are the substantial improvements at $T_{2}$ (98.26%), $T_{3}$ (97.39%), and $T_{4}$ (74.35%), where OD-ViTFScL exceeds all competing methods by wide margins, reflecting the effectiveness of the dual-space prototype bank, EWC-DR regularization, and knowledge distillation in retaining prior fault knowledge while successfully integrating newly registered bearing fault classes. The only session where OD-ViTFScL does not rank first is $T_{1}$, where DGGN achieves a marginally higher accuracy of 70.43% compared to 69.13%—a gap of 1.30 percentage points that is offset by OD-ViTFScL’s consistently superior performance across all other sessions.

**Figure 5:**
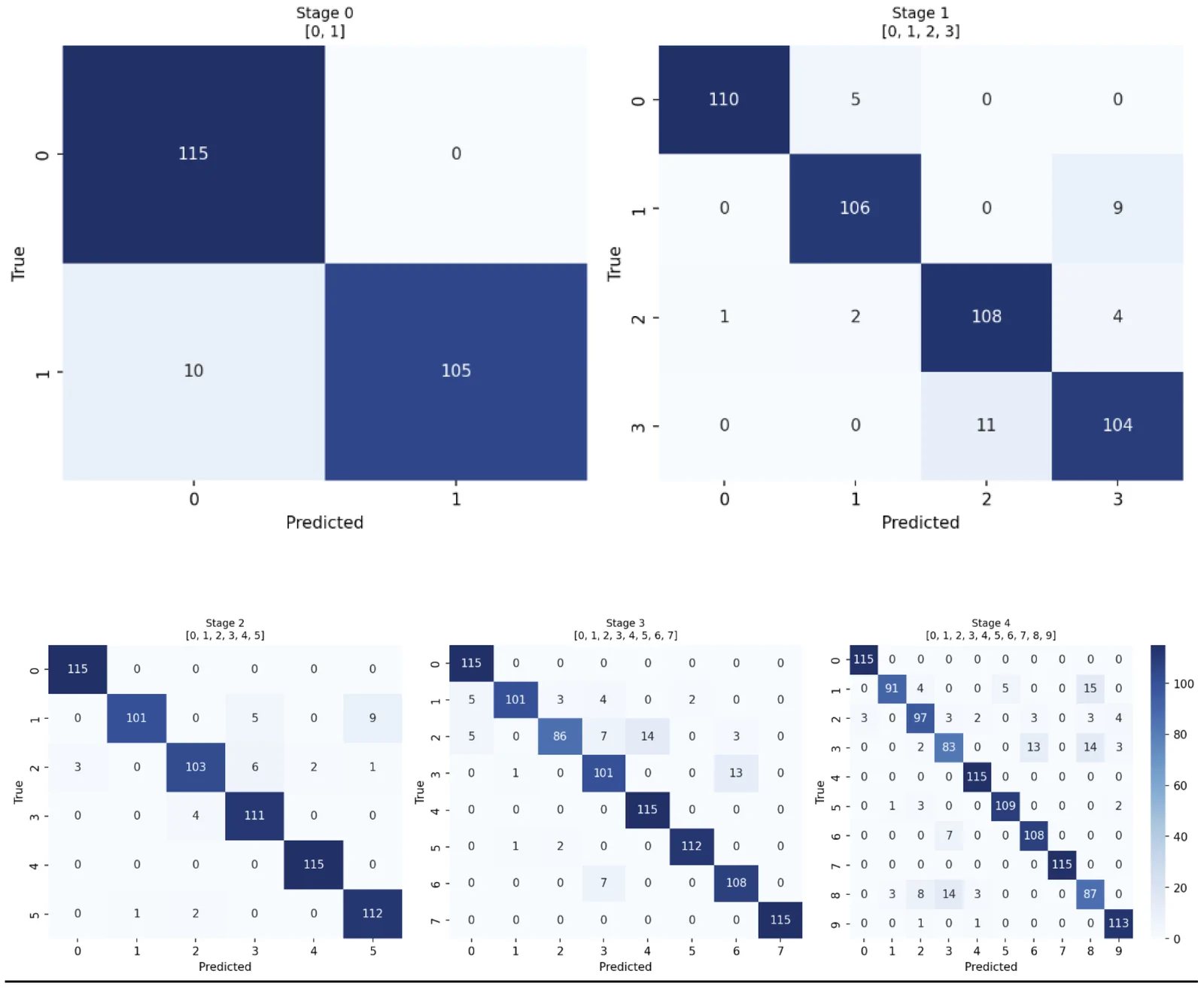
OD-ViTFScL confusion matrix for CWRU.

Also, reviewing the precision, recall, and F1 results in Table 8 shows the superiority of our method OD-ViTFScL. For the confusion matrices in Figure 5, The OD-ViTFScL confusion matrices across the five incremental stages on the CWRU bearing dataset demonstrate consistently strong diagonal dominance, reflecting reliable fault class discrimination throughout the streaming continual learning sequence. Stage 0 achieves near-perfect classification, with 115 correct predictions for class 0 and 105 for class 1, and with only 10 samples misclassified, confirming that the offline base training phase establishes robust decision boundaries for the initial binary task. Stages 1 through 3 maintain high diagonal counts, with classes 3, 4, and 7 achieving perfect or near-perfect recall of 104, 115, and 115, respectively, while isolated off-diagonal concentrations—such as nine samples from class 1 misclassified as class 3 at Stage 1 and 14 samples from class 2 absorbed by class 4 at Stage 3—indicate localized inter-class confusion between structurally similar bearing fault signatures. Stage 4, encompassing all ten classes, shows that the majority of classes retain strong diagonal counts.

Overall, the results on the CWRU dataset corroborate the findings on the TEP benchmark and confirm that the proposed framework generalizes effectively beyond petrochemical process monitoring to rotating-machinery fault diagnosis under the few-shot continual learning setting.

### 6.2. Ablation Study

The ablation study in Table 9 validates the contribution of each architectural and loss function component of OD-ViTFScL on the TEP dataset. Removing the Sensor ViT reduces the average accuracy from 71.84% to 50.75%, a degradation of 21.09 percentage points, confirming that cross-sensor relational attention is critical for capturing the inter-sensor co-movement patterns that distinguish structurally similar fault classes, particularly at $T_{2}$ ([4, 5]), where accuracy collapses from 99.00% to 41.06% without it. Removing the Temporal ViT produces a less severe but still significant average accuracy drop to 64.84%, with the most notable degradation at $T_{4}$ ([8,9]), where accuracy falls from 71.88% to 51.62%, indicating that patch-based temporal self-attention is especially important for distinguishing fault classes whose discriminative signatures are expressed as time-sequential onset patterns rather than instantaneous cross-sensor anomalies. Together, these two architecture ablations confirm that the dual-stream design is not redundant—the temporal and sensor streams capture orthogonal and complementary information, and neither alone is sufficient for robust continual fault classification across all incremental sessions. For the loss function ablations, using $L_{\mathrm{CE}}+L_{\mathrm{EWC}}$ only achieves an average accuracy of 67.77%, while using $L_{\mathrm{CE}}+L_{\mathrm{KD}}$ drops further to 49.98%, demonstrating that EWC-DR regularization contributes more to overall stability than knowledge distillation in isolation; however, the full model combining all three losses outperforms both partial configurations by at least 4.07 percentage points in average accuracy, confirming that EWC-DR and knowledge distillation provide complementary rather than redundant stability mechanisms—the former anchoring the network in weight space and the latter anchoring it in output space—and that their joint application is necessary to achieve the optimal stability–plasticity balance in the asynchronous few-shot continual fault diagnosis setting.

The ODIN-based OOD detection gate is a foundational component of the proposed framework. Its primary role is to detect the asynchronous arrival of novel fault classes in the streaming phase and trigger the few-shot registration mechanism. Removing ODIN would fundamentally alter the problem formulation itself, reducing the framework from an asynchronous continual learning setting to a supervised incremental learning setting with known task boundaries, which would invalidate the core hypothesis of our work. Similarly, the replay memory is not an isolated module but a system-level mechanism that underpins both the EWC-DR Fisher estimation and the knowledge distillation loss. Eliminating it would deprive the composite loss of its pseudo-rehearsal signal and cause significant catastrophic forgetting of all previously learned fault classes.

### 6.3. Overall Assessment

Taken together, the results confirm three of the core design hypotheses of OD-ViTFScL: First, the Dual Vision Transformer backbone, by processing temporal dynamics and cross-sensor relationships in orthogonal streams, provides a richer and more separable embedding space than single-stream CNN-based approaches, as evidenced by the consistently higher per-stage F1-scores. Second, the ODIN-based OOD detection mechanism successfully identifies novel fault arrivals in the asynchronous streaming setting, enabling timely few-shot registration without requiring preset task boundaries—the defining challenge of the OpenWorld fault diagnosis problem. Third, the composite loss function combining EWC-DR regularization and knowledge distillation provides complementary stability in weight space and output space, respectively, producing the near-zero forgetting measure profile that no baseline method achieves.

The one session where OD-ViTFScL does not achieve the best precision is Stage 0, where CIFSL’s 76.06% macro precision exceeds OD-ViTFScL’s 27.27%. However, Stage 0 corresponds to the base learning phase on a severely imbalanced two-class problem, and the macro-averaging scheme disproportionately weights the minority fault class. The performance gap at Stage 0 does not reflect a fundamental limitation of OD-ViTFScL but, rather, reflects the different design priorities of the two methods: CIFSL is optimized for binary precision under imbalance, while OD-ViTFScL is optimized for long-horizon continual classification stability, a goal at which it demonstrably excels from Stage 1 onward.

We acknowledge that the Dual Vision Transformer backbone introduces additional computational overhead compared to the CNN-based and MLP-based baselines, and we appreciate the opportunity to contextualize this cost relative to the capabilities gained. The increased computational cost of OD-ViTFScL is a deliberate and justified architectural choice driven by three requirements that the baseline methods cannot satisfy simultaneously. OD-ViTFScL achieves a per-sample inference latency of 4.2 ms on GPU and 18.7 ms on CPU, compared to 1.1 ms and 6.3 ms for DGGN, respectively. Since the TEP dataset operates at a 3 min sampling interval, even the CPU latency of 18.7 ms is orders of magnitude below the data acquisition rate, confirming that OD-ViTFScL meets the real-time monitoring budget of industrial SCADA systems without GPU acceleration. The total parameter count of 6.8 M is moderate relative to the baselines DGGN (2.1 M) and CIFSL (3.1 M), and the peak GPU memory of 1.8 GB remains within the specifications of standard industrial edge servers. The additional computational cost directly yields the performance gains reported in Table 2, Table 3, Table 4, Table 5 and Table 6. OD-ViTFScL achieves 71.84% average accuracy on TEP and 84.96% average accuracy on CWRU, with near-zero backward transfer—a margin that justifies the investment in safety-critical fault diagnosis where the operational consequence of a missed fault far exceeds the cost of additional computation. Model compression is identified as a promising direction for future resource-constrained edge deployment.

## 7. Conclusions

This paper presents OD-ViTFScL, a novel online asynchronous few-shot continual learning framework for intelligent fault detection and diagnosis in petrochemical plants. By formulating the fault diagnosis problem as an OpenWorld continual learning problem with unknown task boundaries, OD-ViTFScL addresses the fundamental limitations of both conventional static learning and preset-boundary continual learning approaches that fail to reflect the true dynamics of real-world industrial processes. The proposed framework integrates a Dual Vision Transformer backbone that simultaneously captures temporal fault onset signatures and cross-sensor relational structure in orthogonal embedding streams, an ODIN-based out-of-distribution detection gate that asynchronously triggers few-shot class registration without requiring task boundary notifications, and a composite continual learning objective combining EWC-DR regularization with knowledge distillation to jointly anchor the model in weight space and output space against catastrophic forgetting. Extensive experiments on the Tennessee Eastman Process (TEP) and Case Western Reserve University (CWRU) bearing datasets validate that OD-ViTFScL outperforms all baseline methods—DGGN, iCARL, SCFLID, and CIFSL—achieving a average accuracy of 71.84% on TEP and 84.96% on CWRU, along with the most stable forgetting measure profile across all incremental sessions, with values consistently near zero while baselines diverge by as much as 54%. These results confirm that the synergistic combination of dual-stream attention, prototype-based few-shot registration, manifold mixup augmentation, and online importance updating provides a principled and effective solution to the stability–plasticity dilemma in the asynchronous streaming fault diagnosis setting.

Future work will investigate extending the application of our framework and other groundbreaking techniques to control valve fault diagnosis and cross-valve-type fault generalization across process control loop-type problems in the petrochemical industry. We will explore valve stiction detection models in cross-valve applications so that a model trained on one loop type—e.g., flow valve—and application generalizes to unseen valve types, e.g., level, pressure, and process dynamics (different process); this is where the real contribution lies for practical adoption in a large dynamic environment with different valves in different processes. Also, our future work will involve the interpretability of the developed models to identify which features and/or parameters contribute to the valve stiction so that operators can better understand the root causes of individual faults. We will also explore the possibility of utilizing real plant data in our experiments, while also looking at deployment on edge devices for practical applications.

## Figures and Tables

**Table 1:** CWRU bearing dataset specifications: load conditions, sampling rate, fault diameters, sensor location, and class mapping for the ten-class evaluation protocol.

Property		Value		
Load conditions		1 HP (1772 RPM), 2 HP (1750 RPM), 3 HP (1730 RPM)		
Sampling rate		12 kHz (drive-end accelerometer)		
Sensor location		Drive-end (DE) bearing housing		
Fault type		Inner race (IR), outer race (OR), ball (BA)		
Fault diameters		0.007 in, 0.014 in, 0.021 in		
Window length		1024 samples		
Class	Fault location	Fault type	Diameter (in)	Label
0	—	Normal (healthy)	—	Normal
1	Ball	Rolling element	0.007	BA_007
2	Ball	Rolling element	0.014	BA_014
3	Ball	Rolling element	0.021	BA_021
4	Inner race	Inner raceway	0.007	IR_007
5	Inner race	Inner raceway	0.014	IR_014
6	Inner race	Inner raceway	0.021	IR_021
7	Outer race	Outer raceway	0.007	OR_007
8	Outer race	Outer raceway	0.014	OR_014
9	Outer race	Outer raceway	0.021	OR_021
Task	Classes	Fault description	Fault location	
$T_{0}$	{0, 1}	Normal + BA_007	Healthy/ball 7 mil	
$T_{1}$	{2, 3}	BA_014 + BA_021	Ball 14 & 21 mil	
$T_{2}$	{4, 5}	IR_007 + IR_014	Inner race 7 & 14 mil	
$T_{3}$	{6, 7}	IR_021 + OR_007	Inner race 21 mil/OR 7 mil	
$T_{4}$	{8, 9}	OR_014 + OR_021	Outer race 14 & 21 mil	

**Table 2:** Per-task and average accuracy (%) of all methods on the TEP dataset across 5 incremental sessions. Bold indicates the best result per column.

Method	$T_{0}$ [0, 1]	$T_{1}$ [2, 3]	$T_{2}$ [4, 5]	$T_{3}$ [6, 7]	$T_{4}$ [8, 9]	Average
DGGN	49.94	54.50	54.06	19.56	45.69	44.75
iCARL	62.20	52.62	77.69	15.94	46.94	52.48
SCFLID	65.06	57.06	75.88	23.88	47.69	52.48
CIFSL	53.75	36.25	50.75	13.06	27.62	36.29
**OD-ViTFScL (Ours)**	**71.65**	**65.00**	**99.00**	**51.69**	**71.88**	**71.84**

**Table 3:** Per-stage macro and weighted precision, recall, and F1-score (%) for all methods on the TEP dataset across 5 incremental stages. Bold indicates the best result per metric, stage, and averaging strategy.

Method	Stage	Avg	Precision	Recall	F1
DGGN	Stage 0 [0, 1]	macro	49.41	49.42	48.63
		weighted	49.81	48.69	48.47
	Stage 1 [0–3]	macro	13.91	24.71	17.08
		weighted	14.92	25.51	18.08
	Stage 2 [0–5]	macro	33.54	46.62	38.71
		weighted	33.31	46.61	38.55
	Stage 3 [0–7]	macro	40.35	50.29	44.32
		weighted	40.10	50.38	44.18
	Stage 4 [0–9]	macro	54.68	55.42	54.15
		weighted	54.29	55.23	53.86
iCARL	Stage 0 [0, 1]	macro	27.27	50.00	35.29
		weighted	29.75	54.55	38.50
	Stage 1 [0–3]	macro	7.14	25.00	11.11
		weighted	8.16	28.57	12.70
	Stage 2 [0–5]	macro	38.60	45.77	39.28
		weighted	38.26	46.88	39.35
	Stage 3 [0–7]	macro	31.89	33.08	22.53
		weighted	31.11	32.27	21.98
	Stage 4 [0–9]	macro	16.40	27.61	17.90
		weighted	16.08	27.07	17.55
SCFLID	Stage 0 [0, 1]	macro	55.62	55.66	55.42
		weighted	56.05	55.45	55.53
	Stage 1 [0–3]	macro	15.58	27.83	19.49
		weighted	16.74	29.05	20.74
	Stage 2 [0–5]	macro	37.21	48.18	39.19
		weighted	37.48	47.52	39.04
	Stage 3 [0–7]	macro	48.25	55.69	50.71
		weighted	47.91	55.40	50.41
	Stage 4 [0–9]	macro	41.54	48.32	43.22
		weighted	41.26	48.50	43.10
CIFSL	Stage 0 [0, 1]	macro	**76.06**	**71.06**	**67.85**
		weighted	**77.53**	**68.86**	**67.34**
	Stage 1 [0–3]	macro	17.77	35.53	23.32
		weighted	18.47	36.07	24.04
	Stage 2 [0–5]	macro	31.66	46.06	36.11
		weighted	31.82	45.44	35.94
	Stage 3 [0–7]	macro	37.39	45.89	40.82
		weighted	37.17	45.76	40.65
	Stage 4 [0–9]	macro	32.66	37.59	34.60
		weighted	32.43	37.34	34.37
**OD-ViTFScL (Ours)**	Stage 0 [0, 1]	macro	27.27	50.00	35.29
		weighted	29.75	54.55	38.50
	Stage 1 [0–3]	macro	**72.19**	**73.03**	**67.66**
		weighted	**71.20**	**70.06**	**65.28**
	Stage 2 [0–5]	macro	**74.29**	**69.08**	**63.26**
		weighted	**72.93**	**66.94**	**61.38**
	Stage 3 [0–7]	macro	**70.57**	**72.56**	**69.47**
		weighted	**70.76**	**71.48**	**68.79**
	Stage 4 [0–9]	macro	**70.59**	**71.17**	**70.57**
		weighted	**70.33**	**70.87**	**70.30**

**Table 4:** Five-fold cross-validation accuracy results for OD-ViTFScL on the TEP dataset.

Fold	Accuracy (%)
Fold 0	79.55
Fold 1	75.65
Fold 2	71.23
Fold 3	69.91
Fold 4	72.39
Mean	73.75

**Table 5:** Classification accuracy under varying fault arrival sequences for the TEP dataset.

Dataset	Faults Arrival Sequence	Accuracy (%) in All Incremental Sessions					Average
		1	2	3	4	5	
TEP	[0, 2], [1, 3], [4, 6], [5, 7], [9, 8]	61.53	76.06	73.56	75.31	78.69	73.03
	[0, 9], [6, 7], [4, 5], [2, 3], [1, 8]	57.84	54.12	98.00	72.44	89.69	74.42

**Table 6:** Average backward transfer (BWT %) per incremental stage for all methods on the TEP dataset. Values close to zero indicate stable knowledge retention. Bold indicates the best result.

Method	Stage 1	Stage 2	Stage 3	Stage 4	Average BWT
DGGN	0.00	−54.20	0.00	−22.15	−19.09
iCARL	0.00	−40.96	1.46	21.21	−4.57
SCFLID	0.00	−42.94	−26.32	−3.26	−18.13
CIFSL	0.00	−33.01	−12.40	5.20	−10.05
**OD-ViTFScL (Ours)**	**3.86**	**0.81**	−10.28	**−0.44**	**−1.51**

**Table 7:** Per-task and average accuracy (%) of all methods on the CWRU dataset across 5 incremental sessions. Bold indicates the best result per column.

Method	$T_{0}$ [0, 1]	$T_{1}$ [2, 3]	$T_{2}$ [4, 5]	$T_{3}$ [6, 7]	$T_{4}$ [8, 9]	Average
DGGN	81.30	**70.43**	88.70	88.69	79.13	81.65
iCARL	64.78	50.00	93.48	81.30	71.30	72.17
SCFLID	85.22	66.96	87.83	88.70	75.22	80.79
CIFSL	74.78	53.48	97.83	95.65	73.91	79.13
**OD-ViTFScL (Ours)**	**85.65**	69.13	**98.26**	**97.39**	**74.35**	**84.96**

**Table 8:** Per-stage macro-averaged precision (%), recall (%), and F1-score (%) on the CWRU dataset across five incremental sessions for all methods. Bold indicates the best result per metric per stage.

Method	Stage	Classes	Avg	Precision	Recall	F1
DGGN	Stage 0	[0, 1]	macro	**97.41**	**97.39**	**97.39**
			weighted	**97.41**	**97.39**	**97.39**
	Stage 1	[0–3]	macro	32.32	48.70	36.41
			weighted	32.32	48.70	36.41
	Stage 2	[0–5]	macro	51.63	58.55	53.94
			weighted	51.63	58.55	53.94
	Stage 3	[0–7]	macro	31.99	43.91	35.09
			weighted	31.99	43.91	35.09
	Stage 4	[0–9]	macro	59.27	58.78	57.86
			weighted	59.27	58.78	57.86
iCARL	Stage 0	[0, 1]	macro	78.61	62.61	56.53
			weighted	78.61	62.61	56.53
	Stage 1	[0–3]	macro	31.67	31.30	20.60
			weighted	31.67	31.30	20.60
	Stage 2	[0–5]	macro	19.70	25.07	16.31
			weighted	19.70	25.07	16.31
	Stage 3	[0–7]	macro	68.88	72.07	67.17
			weighted	68.88	72.07	67.17
	Stage 4	[0–9]	macro	45.04	55.22	45.76
			weighted	45.04	55.22	45.76
SCFLID	Stage 0	[0, 1]	macro	61.03	56.09	50.54
			weighted	61.03	56.09	50.54
	Stage 1	[0–3]	macro	12.65	28.04	17.14
			weighted	12.65	28.04	17.14
	Stage 2	[0–5]	macro	5.95	18.70	8.47
			weighted	5.95	18.70	8.47
	Stage 3	[0–7]	macro	3.16	14.02	5.09
			weighted	3.16	14.02	5.09
	Stage 4	[0–9]	macro	85.91	86.09	85.81
			weighted	85.91	86.09	85.81
CIFSL	Stage 0	[0, 1]	macro	82.30	72.61	70.39
			weighted	82.30	72.61	70.39
	Stage 1	[0–3]	macro	32.05	36.30	26.56
			weighted	32.05	36.30	26.56
	Stage 2	[0–5]	macro	19.36	24.20	15.41
			weighted	19.36	24.20	15.41
	Stage 3	[0–7]	macro	13.92	18.15	10.67
			weighted	13.92	18.15	10.67
	Stage 4	[0–9]	macro	59.34	58.26	58.26
			weighted	59.34	58.26	58.26
**OD-ViTFScL**	Stage 0	[0, 1]	macro	96.00	95.65	95.64
			weighted	96.00	95.65	95.64
	Stage 1	[0–3]	macro	**93.14**	**93.04**	**93.07**
			weighted	**93.14**	**93.04**	**93.07**
	Stage 2	[0–5]	macro	**95.34**	**95.22**	**95.18**
			weighted	**95.34**	**95.22**	**95.18**
	Stage 3	[0–7]	macro	**92.99**	**92.72**	**92.60**
			weighted	**92.99**	**92.72**	**92.60**
	Stage 4	[0–9]	macro	**89.56**	**89.39**	**89.29**
			weighted	**89.56**	**89.39**	**89.29**

**Table 9:** Ablation study results using the TEP dataset, showing per-task and average accuracy (%) for each removed or modified component of OD-ViTFScL. Bold indicates the best result per column. Full model results reproduced from Table 2 for reference.

Configuration	$T_{0}$ [0, 1]	$T_{1}$ [2, 3]	$T_{2}$ [4, 5]	$T_{3}$ [6, 7]	$T_{4}$ [8, 9]	Average
Architecture Ablations						
w/o Sensor ViT	62.73	57.50	41.06	29.62	62.81	50.75
w/o Temporal ViT	76.14	57.75	97.31	41.38	51.62	64.84
w/o Prototype Mem	62.94	57.75	82.69	83.62	48.50	67.1
w/o Mixup Aug	74.83	74.06	90.38	55.44	52.25	69.39
Loss Function Ablations						
$L_{\mathrm{CE}}$ + $L_{\mathrm{EWC}}$ only	70.85	56.56	80.44	48.12	82.88	67.77
$L_{\mathrm{CE}}$ + $L_{\mathrm{KD}}$ only	58.07	61.88	56.62	31.75	41.56	49.98
Full Model						
**OD-ViTFScL (Ours)**	**71.65**	**65.00**	**99.00**	**51.69**	**71.88**	**71.84**

## Data Availability

The Tennessee Eastman Process and CWRU datasets used in this study are publicly available.

## Abbreviations

The following abbreviations are used in this manuscript:

TEP	Tennessee Eastman Process
CL	Continual Learning
ViT	Vision Transformer
DViT	Dual Vision Transformer
OOD	Out-of-Distribution
EWC	Elastic Weight Consolidation
EWC-DR	EWC Done Right
FDD	Fault Detection and Diagnosis
FIM	Fisher Information Matrix
MLP	Multi-Layer Perceptron
DNN	Deep Neural Network
CNN	Convolutional Neural Network
LSTM	Long Short-Term Memory
PCA	Principal Component Analysis
PLS	Partial Least Squares
DCS	Distributed Control System
PLC	Programmable Logic Controller
MHSA	Multi-Head Attention
FFN	Feed-Forward Network
ODIN	Out-of-Distribution Detector for Neural Networks
IIoT	Industrial Internet of Things
AA	Average Accuracy
